# The Demand for Elective Neurosurgery at a German University Hospital during the First Wave of COVID-19

**DOI:** 10.3390/healthcare8040483

**Published:** 2020-11-13

**Authors:** Rosita Rupa, Benjamin Sass, Maria Alejandra Morales Lema, Christopher Nimsky, Benjamin Voellger

**Affiliations:** Department of Neurosurgery, University Marburg, Baldingerstr, 35033 Marburg, Germany; rupar@med.uni-marburg.de (R.R.); sassb@med.uni-marburg.de (B.S.); mariaalejandra.moraleslema@uk-gm.de (M.A.M.L.); nimsky@med.uni-marburg.de (C.N.)

**Keywords:** neurosurgery, COVID-19, public education, healthcare

## Abstract

Background: Patients’ fear of the coronavirus disease 2019 (COVID-19) may delay inevitable treatment, putting potential benefits at risk. This single-center retrospective study aims to analyze temporal relationships of the first wave of the COVID-19 pandemic in Germany with the number of patients who sought and received elective neurosurgical treatment at a German university hospital. Methods: Daily outpatient numbers (ON) and elective procedures (EP) were recorded at our department between 1 January 2020 and 30 June 2020 (baseline: between 1 January 2019 and 30 June 2019). In patients who received EP, we recorded indication, outcome, and length of stay (LOS). Moving averages of ON (MAON) and of EP were calculated. Data on governmental action taken in response to the pandemic and on coronavirus-positive cases in Germany (CPCG) were superimposed. Exponential and arc tangent curves (ATC) were fitted to the absolute numbers of CPCG. Phase shifts were estimated, and Spearman’s rank correlation coefficient, *rho*, was calculated between the 2020 MAON and the derivative function of the fitted ATC (DFATC). Wilcoxon rank sum served as statistical test. Significance was assumed with *p* values of less than 0.05. Results: ON were significantly decreased in April 2020 as compared to April 2019 (*p* = 0.010). A phase shift between the German lockdown, the DFATC, and the decrease in MAON was not detected, while a phase shift of 10 days between the DFATC and the subsequent increase in MAON was detected. The DFATC was significantly negatively correlated (*rho* = −0.92, *p* < 0.0001) to the MAON until 31 March 2020, and, when shifted by 10 days, the DFATC was significantly negatively correlated (*rho* = −0.87, *p* < 0.0001) to the MAON from 01 April 2020. EP (*p* = 0.023), including the subset of non-oncological EP (*p* = 0.032), were significantly less performed in the first half of 2020 as compared to the first half of 2019. In March and April 2020, we conducted significantly more EP due to motor deficits (*p* = 0.0267, and less), visual disturbances (*p* = 0.0488), and spinal instability (*p* = 0.0012), and significantly less EP due to radicular pain (*p* = 0.0489), as compared to March and April 2019. LOS ranked significantly higher in patients who received cranial or spinal EP in March and April 2020 as compared to March and April 2019 (*p* = 0.0497). Significant differences in outcome were not observed. Conclusion: The beginning of the COVID-19 pandemic was correlated to an immediate and significant decrease in ON, and to a significant decrease in the number of EP performed. The subsequent increase in ON was delayed. Adequate measures to promote timely discharge of patients may become increasingly relevant as the pandemic proceeds. Although we observed a shift in the range of indications towards significantly more EP in patients with neurological deficiencies, care should be taken to avoid potentially deleterious delays of necessary elective treatment in future pandemic situations.

## 1. Introduction

Worldwide, clinicians share the concern that patients’ fear of the COVID-19 pandemic may delay inevitable treatment, and that such delays may put the potential treatment benefits at risk, e.g., in adult patients who are in need of emergency care [1,2], as well as in children [3], or in cancer patients [4]. The psychological burden for cancer patients during the COVID-19 pandemic and the related fear have been analyzed by Musche et al. [5]. Deo et al. [6] have described the challenges to tumor surgery arising from the COVID-19 pandemic and proposed corresponding guidelines, with the dynamics of the pandemic, available resources, patient characteristics, and the stage of the disease being considered as key factors. Wilson et al. [7], and Rizkalla et al. [8] have developed recommendations for triaging spinal surgery during the COVID-19 pandemic. Neurosurgical emergencies at a German university hospital during the COVID-19 pandemic have recently been covered in an interesting report by Hecht et al. [9]. Our single-center retrospective study aims to analyze temporal relationships of the first wave of the COVID-19 pandemic in Germany with the number of patients who, at the same time, sought and received elective neurosurgical treatment at a German university hospital, and to analyze changes in the corresponding range of indications for elective procedures (EP).

## 2. Methods

**Figure 1:**
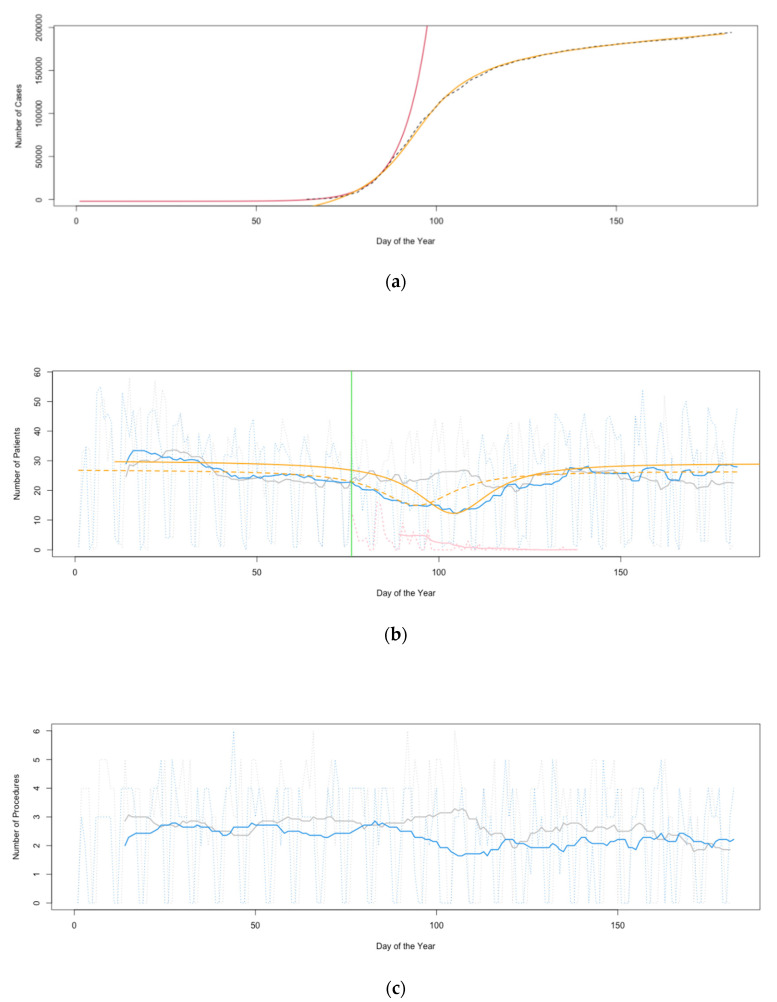

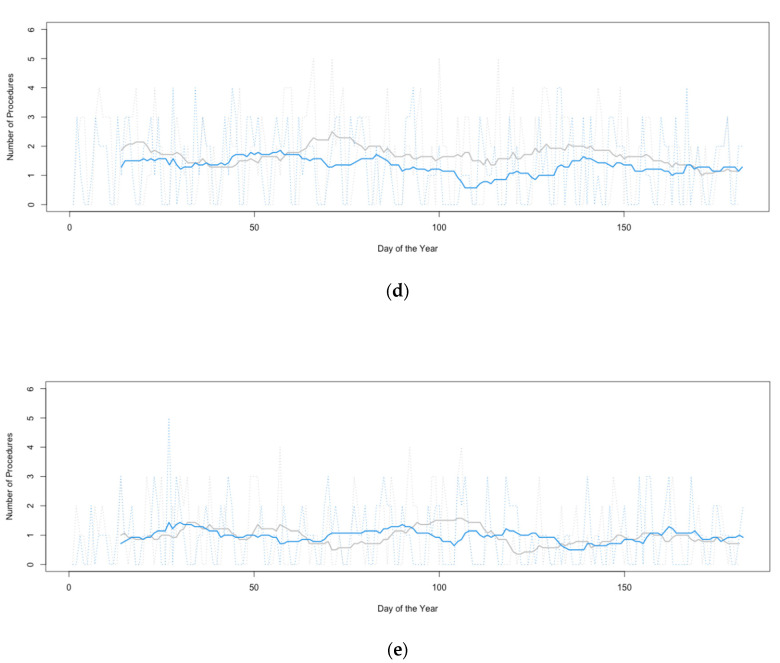
(**a**). Novel coronavirus-positive case numbers in Germany as published by the Robert Koch Institute (RKI) from 4 March 2020 (absolute values; black, dashed). Exponential (red) and arc tangent (ATC; orange) curves were manually fitted to the RKI data; the formulas of the fitted curves are given in the methods section of this paper (Formulas (1) and (2)). (**b**). Daily outpatient numbers (ON; grey, dotted) and moving averages thereof (MAON; grey) in the first half of 2019 as compared to daily ON (blue, dotted) and MAON (blue) in the first half of 2020. ON were significantly decreased in April 2020 as compared to April 2019 (*p* = 0.010, Table 1). Numbers of appointments cancelled between 16 March 2020 and 15 May 2020 explicitly due to the coronavirus 19 disease (COVID-19) pandemic (pink, dotted), and moving averages thereof (pink). Lockdown in Germany due to the COVID-19 pandemic on 16 March 2020 (green). The derivative function of the ATC from Figure 1a (DFATC; orange, dashed; Formula (3) given in the methods section of this paper) was significantly negatively correlated (*rho* = −0.92, *p* < 0.0001) to the MAON from 1 February 2020 until 31 March 2020, and, when shifted by 10 days (orange), the DFATC was significantly negatively correlated to the MAON from 1 April 2020 until 30 June 2020 (*rho* = −0.87, *p* < 0.0001). To allow for a better comparison with the 2020 MAON, the DFATC was flipped upside down with its baseline and its ordinate extension adjusted. (**c**). Daily neurosurgical elective procedures (EP; grey, dotted) and moving averages thereof (grey) in the first half of 2019 as compared to daily EP (blue, dotted) and moving averages thereof (blue) in the first half of 2020. EP were significantly less performed (*p* = 0.023, Table 1) in the first half of 2020 as compared to the first half of 2019. (**d**). Daily neurosurgical non-oncological elective procedures (NEP; grey, dotted) and moving averages thereof (grey) in the first half of 2019 as compared to daily NEP (blue, dotted) and moving averages thereof (blue) in the first half of 2020. NEP were significantly less performed (*p* = 0.032, Table 1) in the first half of 2020 as compared to the first half of 2019. (**e**). Daily neurosurgical oncological elective procedures (OEP; grey, dotted) and moving averages thereof (grey) in the first half of 2019 as compared to daily OEP (blue, dotted) and moving averages thereof (blue) in the first half of 2020. Significant differences in frequencies of OEP between the first halves of both years were not observed.

The Hospital Information System (HIS) was queried for daily outpatient numbers (ON) as scheduled in our appointment calendar and for daily numbers of EP at our neurosurgical department between 1 January 2020 and 30 June 2020. To define a baseline, HIS was queried for daily ON and for daily numbers of EP at our neurosurgical department between 1 January 2019 and 30 June 2019. Cases with rapid progression of neurological deficiencies were not considered elective. EP were dichotomized for oncological and other. Between 16 March 2020 and 15 May 2020, appointments cancelled by patients explicitly due to the pandemic, and the respective diagnosis-related groups (DRGs) were recorded by a nurse. Indications for EP between 1 March 2020 and 30 April 2020 (baseline: between 1 March 2019 and 30 April 2019), as established by our consultants during the outpatient visits, were tabulated. In cases with multiple indications, the main indication for each patient was defined according to the information obtained from the respective operation report, with manifest or impending neurological deficiencies, such as aphasia, paresis, or visual disturbance, considered most important. Outcome and length of hospital stay (LOS) were recorded for all patients who received EP between 1 March 2020 and 30 April 2020 (baseline: between 1 March 2019 and 30 April 2019). Patient data were pseudonymized. 14 day-moving averages of ON (MAON) and of EP were calculated. We superimposed patient data with data on governmental recommendations and decisions in Germany [10], and with data on novel coronavirus-positive cases in Germany (CPCG) as published daily from 4 March 2020 by the Robert Koch Institute (RKI) [11]. An exponential curve for the beginning of the pandemic (1) and an arc tangent curve (ATC) for the remaining first wave of the pandemic (2) were manually fitted by author B.V. to the absolute numbers of CPCG (*d*, time in days from 01 January 2020; Figure 1a) as follows:

(1) $$-2*10^{3}+3.14^{0.125*(d-12)}$$

(2) $$7*(10^{4}+0.1*d^{2}+10^{4}*\arctan(0.08*(d-94)))$$

Phase shifts were estimated, and Spearman’s rank correlation coefficient, *rho,* was calculated between the 2020 MAON and the derivative function of the fitted ATC (DFATC, 3). Chi square, Fisher’s exact, and Wilcoxon rank sum served as statistical tests. Significance was assumed with *p* values of less than 0.05.

(3) $$7*((0.2*d)+800/(1+{\left(0.08*(d-94)\right)}^{2}))$$

Statistical analysis was conducted and figures were created with RStudio Version 1.3.959 (RStudio Inc., Boston, MA, USA) [12] running R Version 4.0.2 (The R Project, Vienna, Austria) [13] and with GIMP Version 2.8.18 [14] on a Mac OS X Version 10.14.6 (Apple Inc., Cupertino, CA, USA).

## 3. Results

Daily ON and EP are depicted in Figure 1b–e. Differences in ON and EP are given in Table 1.

The correlation of the DFATC with the 2020 MAON was estimated using Spearman’s rank correlation coefficient, *rho*. MAON from January 2020 were omitted to diminish the interference of obvious irregularities in ON at the beginning of the year with the attempted regression. We correlated the DFATC with the MAON from 1 February 2020 until 31 March 2020, which resulted in a *rho* value of −0.92 with a *p* value of less than 0.0001. We then shifted the DFATC by 10 days and correlated this curve with the MAON from 1 April 2020 until 30 June 2020, which resulted in a *rho* value of −0.87 with a *p* value of less than 0.0001 (Figure 1b).

To assess whether the significant decrease in ON after the German lockdown on 16 March 2020 (Table 1, Figure 1b) had any clinical relevance, we tabulated the indications for cranial, spinal and peripheral nerve EP as conducted at our department in the months of March and April of the years 2019 and 2020 (Table 2). Significant differences were found for the numbers of procedures indicated due to motor deficits, visual disturbances, and spinal instability, which were performed more frequently in March and April 2020 as compared to March and April 2019, and for procedures indicated due to radicular pain, which were conducted less frequently in March and April 2020 as compared to March and April 2019 (Table 2). Non-significant tendencies to perform less procedures due to low back pain and incidental intracranial lesions, and to conduct less functional procedures, were detected in March and April 2020 as compared to March and April 2019 (Table 2).

In March and April 2019, we conducted 116 cranial EP in 111 patients, and 50 spinal EP in 40 patients (Table 2 and Table 3). In March and April 2020, we performed 103 cranial EP in 88 patients, and 35 spinal EP in 29 patients (Table 2 and Table 3). Outcomes and LOS of these patients are listed in Table 3. The LOS of patients who received cranial or spinal EP between 1 March 2020 and 30 April 2020 ranked significantly higher as compared to the LOS of those who received cranial or spinal EP between 1 March 2019 and 30 April 2019 (Table 3). Significant differences in outcomes were not found (Table 3). The clinical courses of all four patients who, within the observation periods, received peripheral nerve EP (Table 2) were unremarkable.

## 4. Discussion

Governmental action taken in response to the pandemic, and, in particular the recommendations made to postpone elective surgical procedures in German hospitals and to maintain social distancing in Germany from 12 March 2020 onwards, as well as the announcement of German lockdown measures with effect from 16 March 2020 (Figure 1b), probably had a major impact on ON and EP. However, the strong negative correlation of the DFATC with the 2020 MAON (Figure 1b) suggests that patients’ fear of COVID-19 may have been affected by the pace at which the disease had spread and thus contributed to the changes observed in ON and EP. Notably, the beginning of the COVID-19 pandemic in Germany was correlated to an immediate, significant decrease in ON and to a significant decrease in EP, while the increase in MAON towards the end of the first wave of the pandemic was delayed by 10 days (Figure 1b).

It is true that several COVID-19 outbreaks have been observed in German hospitals [15], including our hospital and affecting our patients and staff. Therefore, patients’ fear of being infected with COVID-19 in hospital is understandable to some extent. However, the overall risk of contracting COVID-19 at a German hospital appears to still be low and should be reasonably balanced against the risks inherent in postponing necessary elective treatment [15].

There are several explanations for the significant shift in the range of indications as observed by us and by many of our European colleagues [16] during the first wave of the COVID-19 pandemic (Table 2): Political measures, such as the recommendation to postpone EP, certainly have contributed to this effect. From the neurosurgeon’s perspective, indications for EP basically became much stricter. Many neurosurgical patients who feared to contract COVID-19 at the hospital decided to postpone elective surgery, particularly when they had no neurological deficiencies.

Considering the significantly lower frequency of EP carried out due to radicular pain during the first wave of the pandemic, there is obviously a demand for sufficient conservative pain management as a valid treatment alternative for patients with degenerative diseases of the spine suffering from radicular pain. Regular follow-up visits for patients with incidentalomas, optimized treatment plans for patients eligible to receive functional neurosurgery [17], and early initiation of measures to promote timely discharge of patients, such as individualized discharge planning, and transitional care interventions [18], may become increasingly relevant as the pandemic proceeds. In particular, timely discharge of patients may help to have more beds available for COVID-19 patients during the pandemic [19].

During the first wave of the pandemic, we conducted significantly more EP due to motor deficits, visual disturbances and lesions leading to spinal instability, while statistically significant changes in outcome were not observed in our single-center retrospective analysis. The significant shift in the range of indications at our department did, however, not fully prevent potentially deleterious clinical courses similar to the case reported on in the Supplementary Materials to this article. To avoid delays of necessary treatment in the future, we advocate adequate education of the public and digitalization of medical consultations. We, as well as other authors [4,20,21], suppose that such interventions may help oncological and neurosurgical patients in making an informed decision during the pandemic.

The moving average of the number of appointments cancelled explicitly due to the pandemic is obviously much lower than the gap between the 2019 and 2020 MAON between March and May (Figure 1b), while the number of appointments that were not met primarily due to the pandemic without notifying us of the patients’ decision remains unknown. Thus, in a DRG-driven health system, solely reporting appointments cancelled explicitly due to the pandemic along with their respective DRGs will probably not result in full compensation for the losses of revenue experienced during the first wave of COVID-19 [22,23]. To ensure adequate compensation of each health care provider, governmental reimbursement policies should be tailored to the locally available medical specialties and level of care.

The investigation of other potential reasons for the observed decreases in ON an EP in 2020, such as changes in the team of neurosurgical consultants, or the abundance of moveable feasts in Germany taking place in spring, was not the objective of this study.

## 5. Conclusions

The beginning of the COVID-19 pandemic was correlated to an immediate and significant decrease in ON, and to a significant decrease in the number of EP performed. The subsequent increase in ON was delayed. Adequate measures to promote timely discharge of patients may become increasingly relevant as the pandemic proceeds. Although we observed a shift in the range of indications towards significantly more EP in patients with neurological deficiencies during the first wave of the COVID-19 pandemic, care should be taken to avoid potentially deleterious delays of necessary elective treatment in future pandemic situations.

## Figures and Tables

**Table 1:** Differences between numbers of outpatients and of elective procedures at our neurosurgical department in the first half of 2019 as compared to the first half of 2020.

Numbers of Patients and Procedures	2019	2020	*p* Value
Outpatients, January	931	950	0.773
Outpatients, February	660	695	0.909
Outpatients, March	677	596	0.225
Outpatients, April	748	531	0.010 (*)
Outpatients, May	788	752	1
Outpatients, June	645	815	0.085
Outpatients, January–June	4449	4339	0.601
Non-oncological elective procedures, January–June	302	240	0.032 (*)
Oncological elective procedures, January–June	171	174	0.697
All elective procedures, January–June	473	414	0.023 (*)

The ranks of the daily numbers of outpatients as given in Figure 1b and in the supplement were compared between the corresponding months of the first halves of both years, and between the first halves of both years. The ranks of the daily numbers of elective procedures as given in Figure 1c–e and in the supplement were compared between the corresponding months of the first halves of both years, which did not result in statistically significant differences (results not shown), and between the first halves of both years. Wilcoxon rank sum served as statistical test. (*), statistically significant difference.

**Table 2:** Main indications for elective procedures at our department in March and April 2020 as compared to March and April 2019.

Main Indications	2019	2020	Test	*p* Value
	March and April	March and April		
*Cranial EP*				
Aphasia	6	2	Fisher’s exact	0.287
Functional	18	7	chi square	0.0699 (§)
Hydrocephalus	32	19	chi square	0.1507
Incidentaloma	16	6	chi square	0.0832 (§)
Infectious lesion	2	3	Fisher’s exact	0.6679
Motor deficit	5	15	chi square	0.0167 (*)
Seizures	8	12	chi square	0.3251
Tumor progression	6	8	chi square	0.6124
Visual disturbance	2	8	Fisher’s exact	0.0488 (*)
Other	21	16		
Cranial EP, total	116	103		
*Spinal EP*				
Infectious lesion	13	5	chi square	0.3024
Low back pain	5	0	Fisher’s exact	0.0748 (§)
Motor deficit	7	13	chi square	0.0267 (*)
Myelopathy	3	1	Fisher’s exact	0.64
Radicular pain	18	5	chi square	0.0489 (*)
Spinal claudication	1	2	Fisher’s exact	0.5659
Spinal instability	1	9	Fisher’s exact	0.0012 (*)
Spinal EP, total	50	35		
*Peripheral nerve EP*				
Peripheral nerve EP, Total	3	1		
EP, total	169	139		

EP, elective procedure. (*), *p* less than 0.05, statistically significant difference. (§), *p* less than 0.1.

**Table 3:** Outcome and length of hospital stay of patients who received elective procedures at our department in March and April 2020 as compared to March and April 2019.

Outcome and Length of Stay	2019	2020	Test	*p* Value
	March and April	March and April		
*Patients who received cranial EP*				
Length of stay	8 (2 … 48) days	9 (3 … 77) days	Wilcoxon rank sum	0.0946 (§)
Inhouse mortality	2	1	Fisher’s exact	1
Discharged to palliative care	3	2	Fisher’s exact	1
Discharged to nursing home	4	2	Fisher’s exact	0.6955
Discharged home, dependent on care	5	3	Fisher’s exact	1
Discharged home, independent	71	60	chi square	0.6365
Discharged to rehabilitation facility	26	20	chi square	1
Patients who received cranial EP, total	111	88		
*Patients who received spinal EP*				
Length of stay	9 (3 … 67) days	12 (3 … 111) days	Wilcoxon rank sum	0.2674
Inhouse mortality	2	0	Fisher’s exact	0.5055
Discharged to palliative care	0	0	Fisher’s exact	1
Discharged to nursing home	0	0	Fisher’s exact	1
Discharged home, dependent on care	0	2	Fisher’s exact	0.1731
Discharged home, independent	27	15	chi square	0.2821
Discharged to rehabilitation facility	11	12	chi square	0.3429
Patients who received spinal EP, total	40	29		
*Patients who received cranial or spinal EP*				
Length of stay	8 (2 … 67) days	10 (3 … 111) days	Wilcoxon rank sum	0.0497 (*)

Lengths of stay given as median (min … max) days. EP, elective procedure. (*), *p* less than 0.05, statistically significant difference. (§), *p* less than 0.1.

## Supplementary Materials

The following are available online at https://www.mdpi.com/2227-9032/8/4/483/s1, Supplementary Case report; Supplementary Table S1: Raw data underlying Figure 1.
