# Supplementary material for: The Demand for Elective Neurosurgery at a German University Hospital during the First Wave of COVID-19
Source: Healthcare (Basel). 2020 Nov 13;8(4):483. doi: 10.3390/healthcare8040483 (PMC7712298; doi:10.3390/healthcare8040483)
Supplement: Supplementary file 1 [file healthcare-08-00483-s001.zip › supplementary/supplementary_case_report.pdf]

“This is so lively a case, and contains in it so much of the real condition of the people; that I think, I cannot be too particular in it …”[1]

An 88 years-old lady with a medical history of breast cancer, arterial hypertension, and type II diabetes mellitus had complained of gait disturbance at least for the last 2 years.

In February 2020, the patient fell, and a head trauma resulted. Cranial computerized tomography (CCT) did not show significant findings at that time. Cervical spine computerized tomography (CT), which was routinely performed in the emergency department at our hospital, showed a 20x10x12 mm calcified mass lesion within the spinal canal at the level of T1, suspected to be a meningioma. A magnetic resonance imaging (MRI, suppl. fig. 1a) of the spine was obtained to confirm an intradural mass lesion at the level of T1 occupying more than 50 per cent of the diameter of the spinal canal with consecutive displacement of the spinal cord. We reckoned the patient’s ataxia was consistent with the radiological findings and thus recommended resection of the mass lesion to the patient. The patient, fully capable of making an informed decision, replied that she feared to get infected with coronavirus disease 2019 (COVID-19) at the hospital or during rehabilitation, and she therefore decided to have surgery postponed.

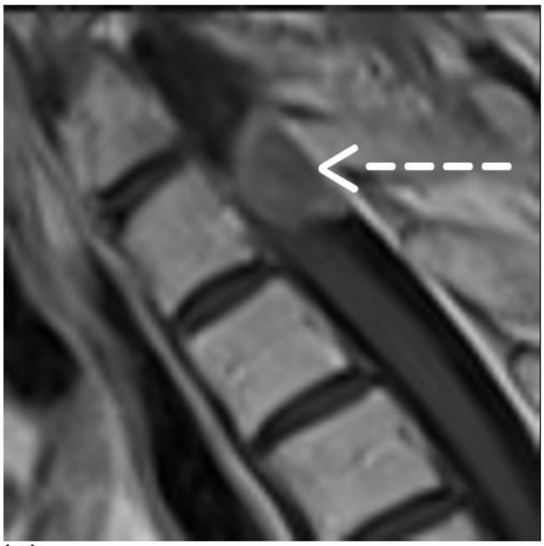

(a)

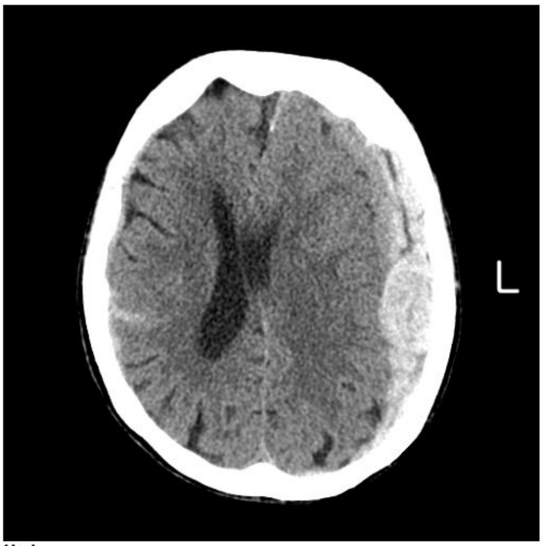

(b)

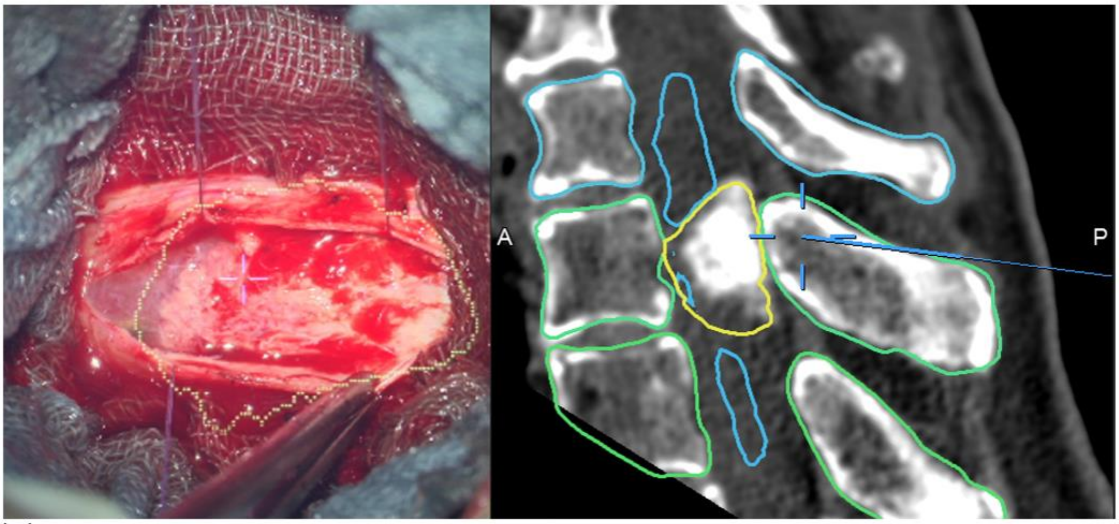

(c)

Supplementary Figure 1. (a) Magnetic resonance imaging (MRI) of the thoracic spine, T1 weighted with contrast enhancement, sagittal view: In February 2020, an 88 years-old lady with a history of gait disturbance was diagnosed with a suspected meningioma at the level of T1 (arrow). Surgical removal of the symptomatic intradural mass lesion was recommended. The lady feared to get infected with coronavirus 2019 disease (COVID-19) at the hospital; she therefore decided to have surgery postponed. (b) Cranial computerized tomography (CCT), axial view: 4½ months later, the same patient, due to progressive gait disturbance, fell at home and subsequently developed rapid clouding of consciousness and a right-sided hemiparesis. She was diagnosed with a massive acute subdural hematoma on the left, which we removed by urgent surgical intervention. (c) Intraoperative imaging: The intradural mass lesion at the level of T1 was resected 9 days later with the help of spinal navigation and intraoperative CT; the neuropathologist confirmed a meningioma of World Health Organization (WHO) grade II. The patient recovered well thereafter.

She agreed, however, to regular outpatient follow-ups. The follow-up MRI in June 2020 did not show tumor growth, while the patient complained of increasing neck pain and gait disturbance. Still, she refused surgical removal of the intradural mass lesion at the level of T1.

About 2 weeks later, the patient was admitted to the emergency department of our hospital after falling at home at around midnight. She complained about severe headache, nausea and amnesia. She had developed clouding of consciousness and a right-sided hemiparesis. The CCT (suppl. fig. 1b) showed a massive acute subdural hematoma on the left, up to 22 mm in width, resulting in a 10 mm midline shift to the right. Moreover, a traumatic subarachnoid hemorrhage on the right frontal lobe and a small fracture of the floor of the left orbit with hemorrhage in the left maxillary sinus were diagnosed.

The acute subdural hematoma was promptly evacuated, and the patient recovered well after surgery. The hemiparesis subsided, and she became again awake and fully oriented. She now agreed to have the tumor removed during the same hospital stay. This procedure was performed via a C7, T1 laminectomy with the help of spinal navigation and intraoperative CT (suppl. fig. 1c) at day 9 after admission. The neuropathologist diagnosed a meningioma of World Health Organization (WHO) grade II. Postoperatively, there were no new neurological deficiencies. The patient was discharged to a neuro-geriatric rehabilitation facility 5 days after tumor surgery.

[1]Daniel Defoe, "A Journal of the Plague Year", first published in 1722
