# Supplementary material for: The Demand for Elective Neurosurgery at a German University Hospital during the First Wave of COVID-19
Source: Healthcare (Basel). 2020 Nov 13;8(4):483. doi: 10.3390/healthcare8040483 (PMC7712298; doi:10.3390/healthcare8040483)
Supplement: Supplementary file 1 [file healthcare-08-00483-s001.zip › supplementary/supplementary_table_s1_page_4.pdf]

cranial_los_2020	cranial_los_2019	spinal_los_2020	spinal_los_2019	los_2020	los_2019
5	5	5	7	5	5
5	5	6	3	5	5
6	15	6	14	6	15
61	3	35	3	61	3
6	10	19	15	6	10
7	7	111	16	7	7
8	6	27	3	8	6
3	8	17	24	3	8
5	42	8	47	5	42
14	3	5	11	14	3
18	4	25	8	18	4
34	5	4	55	34	5
5	5	3	12	5	5
6	7	3	35	6	7
6	14	10	16	6	14
6	30	8	8	6	30
4	6	36	36	4	6
3	4	11	10	3	4
8	4	17	6	8	4
8	8	7	20	8	8
23	4	28	15	23	4
33	19	21	8	33	19
6	8	16	18	6	8
9	10	4	3	9	10
9	36	36	3	9	36
19	12	17	7	19	12
30	7	32	5	30	7
13	5	7	3	13	5
21	14	12	3	21	14
18	15		3	18	15
5	7		7	5	7
7	11		31	7	11
6	4		4	6	4
16	7		67	16	7
6	11		10	6	11
20	25		7	20	25
5	6		4	5	6
7	11		22	7	11
11	9		20	11	9
44	6		4	44	6
7	8			7	8
18	8			18	8
5	12			5	12
27	2			27	2
10	11			10	11
13	5			13	5
11	3			11	3
6	5			6	5
4	5			4	5
6	15			6	15
5	4			5	4
8	3			8	3
5	6			5	6
13	14			13	14
13	9			13	9

6	5	6	5
8	39	8	39
49	13	49	13
23	25	23	25
6	6	6	6
9	20	9	20
8	35	8	35
21	6	21	6
31	3	31	3
46	30	46	30
11	6	11	6
77	24	77	24
14	10	14	10
30	22	30	22
54	22	54	22
3	29	3	29
29	38	29	38
23	24	23	24
30	3	30	3
25	13	25	13
32	15	32	15
7	5	7	5
67	30	67	30
15	10	15	10
4	8	4	8
4	48	4	48
13	36	13	36
14	15	14	15
12	4	12	4
4	28	4	28
4	17	4	17
3	4	3	4
3	6	3	6
	8	5	8
	4	6	4
	21	6	21
	3	35	3
	13	19	13
	19	111	19
	13	27	13
	3	17	3
	3	8	3
	3	5	3
	14	25	14
	2	4	2
	3	3	3
	11	3	11
	4	10	4
	5	8	5
	13	36	13
	12	11	12
	3	17	3
	5	7	5
	4	28	4
	18	21	18
	4	16	4

4	7
36	3
17	14
32	3
7	15
12	16
	3
	24
	47
	11
	8
	55
	12
	35
	16
	8
	36
	10
	6
	20
	15
	8
	18
	3
	3
	7
	5
	3
	3
	3
	7
	31
	4
	67
	10
	7
	4
	22
	20
	4
