# Supplementary material for: The Demand for Elective Neurosurgery at a German University Hospital during the First Wave of COVID-19
Source: Healthcare (Basel). 2020 Nov 13;8(4):483. doi: 10.3390/healthcare8040483 (PMC7712298; doi:10.3390/healthcare8040483)
Supplement: Supplementary file 1 [file healthcare-08-00483-s001.zip › supplementary/supplementary_table_s1_page_1.pdf]

day	outpatients_2019	outpatients_2020	cancellations_2020
1	2	1	NA
2	33	22	NA
3	34	35	NA
4	34	0	NA
5	0	3	NA
6	1	53	NA
7	49	55	NA
8	51	44	NA
9	30	46	NA
10	41	43	NA
11	25	4	NA
12	3	2	NA
13	1	53	NA
14	41	45	NA
15	58	38	NA
16	28	47	NA
17	45	35	NA
18	48	0	NA
19	1	3	NA
20	0	45	NA
21	41	47	NA
22	57	47	NA
23	39	38	NA
24	54	32	NA
25	50	4	NA
26	5	4	NA
27	4	42	NA
28	40	42	NA
29	46	46	NA
30	31	36	NA
31	39	38	NA
32	32	2	NA
33	1	1	NA
34	1	39	NA
35	32	31	NA
36	33	35	NA
37	25	39	NA
38	36	24	NA
39	28	5	NA
40	2	1	NA
41	1	32	NA
42	38	32	NA
43	34	31	NA
44	31	41	NA
45	35	24	NA
46	32	4	NA
47	4	0	NA
48	4	40	NA
49	33	44	NA
50	36	26	NA
51	32	33	NA
52	18	35	NA
53	23	4	NA
54	3	2	NA
55	2	36	NA

56	28	33	NA
57	44	36	NA
58	39	33	NA
59	33	31	NA
60	26	1	NA
61	3	1	NA
62	2	31	NA
63	26	38	NA
64	39	33	NA
65	27	28	NA
66	22	23	NA
67	3	3	NA
68	1	1	NA
69	32	33	NA
70	44	31	NA
71	27	36	NA
72	30	31	NA
73	33	27	NA
74	1	1	NA
75	5	2	NA
76	41	32	13
77	36	23	8
78	24	28	3
79	37	13	3
80	37	22	4
81	24	2	0
82	1	2	0
83	2	23	16
84	25	18	15
85	30	19	6
86	30	27	3
87	37	21	3
88	26	1	0
89	3	0	0
90	3	21	9
91	36	25	5
92	40	13	2
93	29	15	6
94	40	23	3
95	30	0	0
96	1	0	0
97	4	23	7
98	36	26	0
99	44	15	0
100	33	27	0
101	43	1	0
102	26	2	0
103	4	3	0
104	3	2	0
105	36	20	2
106	45	24	0
107	31	27	0
108	37	21	3
109	4	2	0
110	3	0	0
111	1	30	2

112	0	39	0
113	33	29	0
114	32	31	1
115	36	27	0
116	33	1	0
117	3	2	0
118	1	35	0
119	41	39	0
120	43	25	0
121	7	29	0
122	43	0	0
123	34	5	0
124	4	4	0
125	1	44	0
126	38	34	0
127	42	29	0
128	31	29	0
129	35	34	0
130	36	1	0
131	1	1	0
132	1	46	0
133	40	44	0
134	40	42	1
135	40	35	0
136	30	40	0
137	28	2	0
138	7	1	0
139	3	39	NA
140	34	46	NA
141	35	34	NA
142	40	4	NA
143	34	27	NA
144	35	2	NA
145	4	0	NA
146	1	44	NA
147	43	45	NA
148	42	40	NA
149	24	37	NA
150	2	39	NA
151	33	1	NA
152	2	3	NA
153	3	4	NA
154	45	45	NA
155	40	35	NA
156	34	54	NA
157	29	35	NA
158	26	4	NA
159	1	2	NA
160	2	35	NA
161	1	43	NA
162	52	34	NA
163	34	1	NA
164	32	31	NA
165	25	4	NA
166	0	0	NA
167	4	47	NA

168	34	50	NA
169	42	37	NA
170	29	29	NA
171	6	34	NA
172	33	3	NA
173	3	3	NA
174	3	48	NA
175	31	43	NA
176	36	42	NA
177	29	28	NA
178	37	33	NA
179	30	3	NA
180	0	2	NA
181	2	38	NA
182		48	NA
