# Supplementary material for: The Demand for Elective Neurosurgery at a German University Hospital during the First Wave of COVID-19
Source: Healthcare (Basel). 2020 Nov 13;8(4):483. doi: 10.3390/healthcare8040483 (PMC7712298; doi:10.3390/healthcare8040483)
Supplement: Supplementary file 1 [file healthcare-08-00483-s001.zip › supplementary/supplementary_table_s1_page_3.pdf]

day	confirmed_covid_cases_RKI	gov_action_2020
1	NA	NA
2	NA	NA
3	NA	NA
4	NA	NA
5	NA	NA
6	NA	NA
7	NA	NA
8	NA	NA
9	NA	NA
10	NA	NA
11	NA	NA
12	NA	NA
13	NA	NA
14	NA	NA
15	NA	NA
16	NA	NA
17	NA	NA
18	NA	NA
19	NA	NA
20	NA	NA
21	NA	NA
22	NA	NA
23	NA	NA
24	NA	NA
25	NA	NA
26	NA	NA
27	NA	NA
28	NA	NA
29	NA	NA
30	NA	NA
31	NA	NA
32	NA	NA
33	NA	NA
34	NA	NA
35	NA	NA
36	NA	NA
37	NA	NA
38	NA	NA
39	NA	NA
40	NA	NA
41	NA	NA
42	NA	NA
43	NA	NA
44	NA	NA
45	NA	NA
46	NA	NA
47	NA	NA
48	NA	NA
49	NA	NA
50	NA	NA
51	NA	NA
52	NA	NA
53	NA	NA
54	NA	NA
55	NA	NA

56	NA	NA	
57	NA	NA	
58	NA	NA	
59	NA	NA	
60	NA	NA	
61	NA	NA	
62	NA	NA	
63	NA	NA	
64		262	0
65		400	0
66		639	0
67		795	0
68		902	0
69		1139	0
70		1296	0
71		1567	0
72		2369	1
73		3062	0
74		3795	0
75		4838	0
76		6012	1
77		7156	0
78		8198	0
79		10999	0
80		13957	0
81		16662	0
82		18610	0
83		22672	0
84		27436	0
85		31554	0
86		36508	0
87		42288	0
88		48582	0
89		52547	0
90		57298	0
91		61913	0
92		67366	0
93		73522	0
94		79696	0
95		85778	0
96		91714	0
97		95391	0
98		99225	0
99		103228	0
100		108202	0
101		113525	0
102		117658	0
103		120479	0
104		123016	0
105		125098	0
106		127584	0
107		130450	0
108		133830	0
109		137439	0
110		139897	0
111		141672	0

112	143457	0
113	145694	0
114	148046	0
115	150383	0
116	152438	0
117	154175	0
118	155193	0
119	156337	0
120	157641	0
121	159119	0
122	160758	0
123	161703	0
124	162496	0
125	163175	0
126	163860	0
127	164807	0
128	166091	0
129	167300	0
130	168551	0
131	169218	0
132	169575	0
133	170508	0
134	171306	0
135	172239	0
136	173152	0
137	173772	0
138	174355	0
139	174697	0
140	175210	0
141	176007	0
142	176752	0
143	177212	0
144	177850	0
145	178281	0
146	178570	0
147	179002	0
148	179364	0
149	179717	0
150	180458	0
151	181196	0
152	181482	0
153	181805	0
154	182028	0
155	182370	0
156	182764	0
157	183271	0
158	183678	0
159	183979	0
160	184193	0
161	184543	0
162	184861	0
163	185416	0
164	185674	0
165	186022	0
166	186269	0
167	186461	0

168	186839	0
169	187184	0
170	187764	0
171	188534	0
172	189135	0
173	189822	0
174	190359	0
175	190862	0
176	191449	0
177	192079	0
178	192556	0
179	193243	0
180	193449	0
181	193761	0
182	194259	0
