# Supplementary material for: The Demand for Elective Neurosurgery at a German University Hospital during the First Wave of COVID-19
Source: Healthcare (Basel). 2020 Nov 13;8(4):483. doi: 10.3390/healthcare8040483 (PMC7712298; doi:10.3390/healthcare8040483)
Supplement: Supplementary file 1 [file healthcare-08-00483-s001.zip › supplementary/supplementary_table_s1_page_2.pdf]

day	all_ep_2019	all_ep_2020	onco_ep_2019	onco_ep_2020
1	0	0	0	0
2	4	3	2	0
3	4	2	1	1
4	4	0	1	0
5	0	0	0	0
6	0	3	0	2
7	5	3	2	0
8	5	3	1	1
9	5	3	2	1
10	4	3	1	1
11	4	0	1	0
12	0	0	0	0
13	0	4	0	1
14	5	4	3	3
15	3	4	1	1
16	3	4	0	1
17	4	3	1	2
18	4	0	0	0
19	0	0	0	0
20	0	3	0	1
21	4	3	3	1
22	3	4	2	1
23	4	4	0	3
24	3	5	1	2
25	5	0	3	0
26	0	0	0	0
27	0	5	0	5
28	4	4	2	0
29	4	3	1	3
30	5	3	3	2
31	3	3	2	1
32	5	0	3	0
33	0	0	0	0
34	0	4	0	0
35	3	2	2	1
36	3	4	0	1
37	2	4	0	2
38	3	3	3	1
39	3	0	1	0
40	0	0	0	0
41	0	3	0	2
42	4	4	2	1
43	4	4	1	3
44	3	6	1	2
45	3	3	1	0
46	5	0	1	0
47	0	0	0	0
48	0	4	0	1
49	5	4	3	1
50	5	3	3	1
51	4	4	3	1
52	4	3	1	2
53	3	0	1	0
54	0	0	0	0
55	0	3	0	1

56	3	4	1	1
57	5	2	4	0
58	4	4	0	2
59	4	4	0	1
60	4	0	0	0
61	0	0	0	0
62	0	4	0	1
63	4	3	1	2
64	5	3	2	1
65	4	3	0	1
66	6	3	1	1
67	2	0	1	0
68	0	0	0	0
69	0	2	0	2
70	4	4	2	3
71	5	3	0	1
72	2	5	0	2
73	4	4	1	1
74	4	0	0	0
75	0	0	0	0
76	0	4	0	1
77	4	4	3	2
78	4	4	2	1
79	4	4	1	1
80	3	4	0	2
81	4	0	1	0
82	0	0	0	0
83	0	4	0	2
84	4	2	2	2
85	4	4	2	3
86	4	4	0	2
87	4	3	3	2
88	4	0	2	0
89	0	0	0	0
90	0	2	0	2
91	4	4	2	1
92	6	4	4	1
93	3	4	2	0
94	5	1	2	0
95	4	0	0	0
96	0	0	0	0
97	0	3	0	2
98	5	2	3	1
99	3	3	3	2
100	5	4	0	2
101	5	0	3	0
102	4	0	2	0
103	0	0	0	0
104	0	0	0	0
105	6	3	3	3
106	5	3	4	2
107	4	4	1	3
108	4	2	1	1
109	0	0	0	0
110	0	0	0	0
111	0	3	0	0

112	0	2	0	0
113	4	4	1	3
114	3	2	1	1
115	3	3	1	1
116	5	0	0	0
117	0	0	0	0
118	0	3	0	3
119	3	5	0	2
120	5	3	1	2
121	0	4	0	2
122	4	0	1	0
123	3	0	1	0
124	0	0	0	0
125	0	2	0	1
126	4	1	1	0
127	4	4	3	1
128	4	2	0	1
129	5	3	1	1
130	3	0	0	0
131	0	0	0	0
132	0	5	0	1
133	5	4	2	0
134	3	1	0	1
135	4	3	1	1
136	3	3	1	0
137	4	0	2	0
138	0	0	0	0
139	0	4	0	1
140	3	3	1	3
141	2	4	0	1
142	3	0	1	0
143	5	2	1	1
144	5	0	2	0
145	0	0	0	0
146	0	5	0	2
147	4	3	3	0
148	4	3	2	1
149	5	3	1	1
150	0	4	0	2
151	4	0	1	0
152	0	0	0	0
153	0	0	0	0
154	4	3	2	3
155	4	3	2	0
156	4	4	1	3
157	4	4	1	3
158	2	0	1	0
159	0	0	0	0
160	0	4	0	1
161	0	4	0	2
162	4	5	2	3
163	5	0	3	0
164	3	3	1	0
165	3	0	1	0
166	0	0	0	0
167	0	4	0	0

168	2	3	0	3
169	2	2	1	1
170	4	3	2	1
171	0	2	0	1
172	3	0	1	0
173	0	0	0	0
174	0	3	0	2
175	3	4	2	2
176	4	3	2	1
177	3	3	1	1
178	3	4	0	1
179	2	0	1	0
180	0	0	0	0
181	0	3	0	1
182		4		2
